# Temporal profiling with ultra-deep RRBS sequencing reveals the relative rarity of stably maintained methylated CpG sites in human cells: Rarity of stably maintained methylated sites in human cells

**DOI:** 10.3724/abbs.2022185

**Published:** 2022-12-26

**Authors:** Jianing Wang, Yulan Qin, Yani Kang, Xinhui Li, Yuan Wang, Hua Li, Daniel M. Czajkowsky, Zhifeng Shao

**Affiliations:** State Key Laboratory for Oncogenes and Bio-ID Center School of Biomedical Engineering Shanghai Jiao Tong University Shanghai 200240 China

Methylation of cytosine within cytosine-guanine (CpG) sites is one of the most common forms of epigenetic regulation of genomic processes in mammals [1]. As such, faithful transmission of CpG methylation following DNA replication is believed to be essential for the maintenance of cell phenotype and function [2]. However, it is also known that DNA methylation is a dynamic process at a number of CpG sites during cell passaging. In fact, partial methylation is quite common at the population level, implying that there is considerable heterogeneity in methylation at many CpG sites among individual cells in the population. Thus, it is possible that only a portion of all CpG sites must remain methylated in all of the cells in the population to maintain the phenotypic identity and functional state of each cell. However, to date, it remains largely unknown what proportion of CpG sites in the genome are stably maintained in methylation during cell passaging owing to the relatively low depth of sequencing of most datasets and the absence of rigorous, well-controlled experimental conditions to examine this specific issue. In this study, we sought to identify CpG sites whose methylation is stably maintained during cell passaging on a global scale and explore their potential implications and possible clinical applications.

Until now, most studies that examined CpG methylation only measured methylation levels at a single time point, without comparing the levels between different generations, which makes it impossible to identify stably transmitted CpG methylation sites in all cells during passaging. Another weakness in the majority of previous studies is the relatively low sequencing depth when investigating DNA methylation levels at single-base resolution. Since we aimed to identify the fully methylated sites (that is, in all of the cells in the population), it was imperative to obtain a high sequencing depth, as the accuracy for methylation quantification is proportional to this depth. Thus, we employed reduced representation bisulfite sequencing (RRBS), which includes approximately 10% of all CpG sites in the human genome, as a low-cost alternative to examine this essential issue at high sequencing depth [3].

To explore the fidelity of methylation in a range of different cell types, we cultivated three different types of cultured cells (ARPE-19, SW1353, and Jurkat; triplicates for each) and compared their CpG methylation after the 20 ^th^ and 30 ^th^ generations in culture with RRBS. We then examined sites where full methylation was stably maintained from the 20 ^th^ to 30 ^th^ generations. To avoid methylation heterogeneity resulting from differences between different phases of the cell cycle ( Supplementary Materials), we synchronized cells to the G0 phase by serum starvation for 24 h before collecting cells in the 20 ^th^ and 30 ^th^ generations for RRBS analysis. The cells were then collected, and the DNA was extracted using the Axygen Genomic DNA Miniprep Kit (Axygen, Suzhou, China). We then performed RRBS using the extracted DNA, together with a methylated spike-in and an unmethylated spike-in, to determine the conversion error rate of bisulfite conversion, which are critical to assess the full methylation status. High-throughput sequencing was conducted on an Illumina NovaSeq 6000 (Illumina, San Diego, USA) with paired-end 150 bp as the sequencing mode, generating 18 sets of data (triplicates for each cell type at each time point).

Trim Galore was used to perform adapter trimming and quality control on the raw data, followed by the use of Bismark to perform reads alignment and to calculate the methylation level for every CpG site covered by our data ( Supplementary Materials). The proportion of the fragment distribution of our data in the 120 bp to 220 bp region ranged from 62% to 81% (average 72%), and the mapping efficiency ranged from 59% to 76% (average 67%, Supplementary Table S1), which are largely consistent between the biological triplicates. In addition, the bisulfite conversion rate was between 99.0% and 99.3%, and the inappropriate conversion rate was less than 2.5%, which is within the error range of the EZ Gold kit [4]. Given the high consistency between the triplicates ( Supplementary Table S1), we merged each of the triplicate data (that is, each of those obtained at the 20 ^th^ and 30 ^th^ generation) to finally obtain an exceptionally high read coverage (more than 100-fold) for many CpG sites in our dataset.

To verify the quality of our RRBS data, we compared our ARPE-19 and Jurkat data with previously published RRBS data. We subsampled our ARPE-19 and Jurkat RRBS data to the much lower sequencing depth of the published data and then compared the methylation of CpG sites with depths of no less than 10-fold in the two cell types. Overall, we found excellent agreement between our data and previously published work in terms of the number and proportion of methylated CpG sites ( Supplementary Table S2). To make our subsequent analysis more reliable, we filtered out the CpG sites with less than 100-fold read coverage within the six sets of merged data. We then identified CpG sites with methylation levels of 100%, 50%, and 0% and classified the remaining CpG sites as dynamic methylation sites ( Supplementary Materials; Supplementary Table S3), although we note that the latter may also reflect variations resulting from multiple sub-clones within the culture. We also note that, unlike the 100% and 0% methylated sites that reflect sites with truly homogeneous methylation status in all cells, the 50% methylated sites may reflect stable differences between alleles in all cells or differences between two dominant sub-clones. Thus, in this work, we only included these sites in our analysis to compare their maintenance stability with the fully methylated regions but did not investigate their features more deeply.

Interestingly, for the 50% and 0% CpG sites, the vast majority remained the same between the 20 ^th^ and 30 ^th^ generations, with only a small percentage that were different ( Table 1). By contrast, the 100% methylation sites varied considerably between the 20 ^th^ generation and 30 ^th^ generation (ranging from 47% to 66%), indicating a relatively low methylation fidelity for these sites between generations ( Table 1; Supplementary Figure S1). This observation in particular demonstrates the necessity to compare sites at different generations to enable the correct identification of the sites whose methylation status is truly maintained.

**Table:** **Table 1** Classification of high-fidelity CpG sites in ARPE-19, Jurkat, and SW1353 cells

CpG sites(≥100×)	ARPE-19	Jurkat	SW1353
ML=1	ML=0.5	ML=0	ML=1	ML=0.5	ML=0	ML=1	ML=0.5	ML=0
G20	313457	41922	231851	144948	46482	192046	35511	33009	270669
G30	150010	41942	232886	76749	46565	196297	34554	33047	322316
G20∩G30	122868	41705	196276	49137	46123	166546	18813	32499	230500

Note: G20, the 20 ^th^ generation; G30, the 30 ^th^ generation; G20∩G30, sites with stably maintained population-level methylation status between the 20 ^th^ and 30 ^th^ generations; ML: methylation level; ML=1, 100% methylation sites; ML=0.5, 50% methylation sites; ML=0, 0% methylation sites.

Thus, contrary to commonly held beliefs, this analysis reveals that there is only a small set of fully methylated sites that are faithfully maintained during passaging within the human genome. That is, from among the many sites with at least some methylation (one sequenced read) measured at the population level that would typically be considered methylated with single-time point measurements, it is only this small subset that is stably maintained as methylated in each and every cell cycle in all of the cells in the population. Although the RRBS analysis includes only approximately 10% of the CpG sites in the genome, these results suggest that only 2% to 9% of all methylated CpGs in the human genome are of this status. Given the importance of methylation to the phenotypic identity of a cell [2] and the significant paucity of these truly stably maintained methylated sites, it is plausible to assume that these sites are particularly important in these cells. To explore these possibilities, we examined their distribution within a number of annotated genomic features. We found that the majority (63% to 68%) were located in annotated gene bodies for all three cell types ( Supplementary Figure S2), suggesting that they may play a role in transcriptional regulation. Consistent with this, previous work has suggested that higher DNA methylation levels in the gene body could increase transcriptional activity [5], and further, other work has indicated that loss of gene body DNA methylation may reduce transcription elongation or splicing efficiency. The reasons to retain the methylation of only a small portion of these sites in these regions are indeed intriguing and require further investigation.

A particularly important genomic feature often discussed in the context of CpG methylation is the so-called CpG island (CGI), which is an extended genomic locus that exhibits a high GC content and a large density of CpG sites [6]. Unexpectedly, during an initial inspection of the 100% methylated sites within many CGIs in ARPE-19 cells, we observed a notable enrichment near the CGI borders ( Figure 1A). We confirmed this enrichment genome-wide among all CGIs that contained 100% methylated sites in these cells ( Figure 1B). We also observed this enrichment at the CGI border in Jurkat cells ( Supplementary Figure S3A) as well as in SW1353 cells ( Supplementary Figure S3B). We noted that the breadth of the enrichment at the CGI border is ~150 bp in ARPE-19 cells, which is approximately the size of a nucleosome ( Figure 1B; Supplementary Materials). We thus speculated that at least a contributing factor for the enrichment of these fully methylated sites at these borders was a likewise enrichment of nucleosomes at these locations. Hence, we examined nucleosome occupancy in these cells by performing MNase-seq ( Supplementary Materials). Indeed, we observed a significant enrichment of nucleosomes at precisely the same position near the CGI border as the enrichment of the fully methylated sites ( Figure 1C; Supplementary Materials). Thus, it is possible that part of the mechanism underlying the high fidelity of these particular fully methylated sites involves a likewise high fidelity of nucleosome occupancy at these locations, an intriguing possibility for future work.

**Figure 1:**
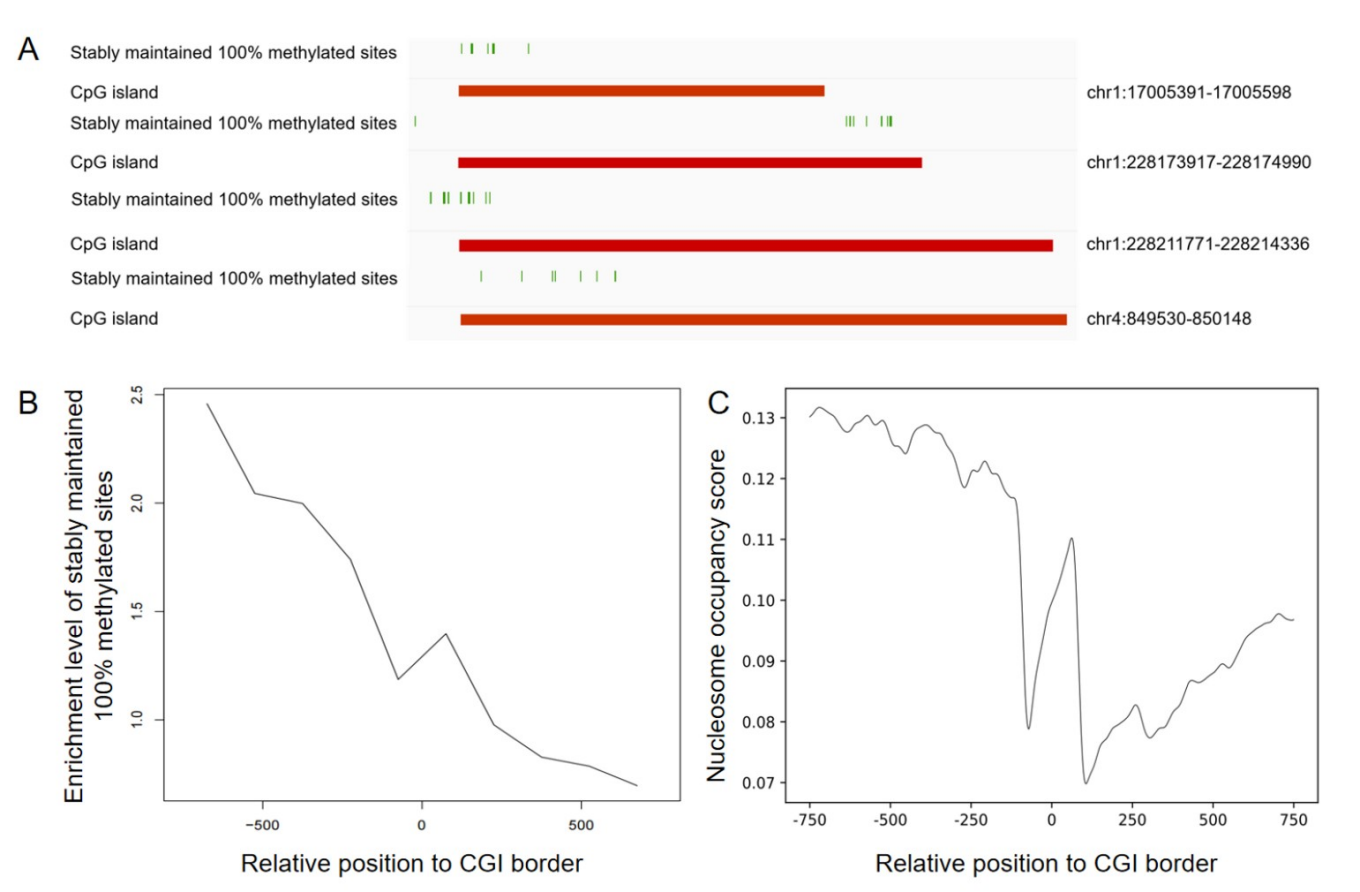
Figure 1 Enrichment of stably maintained 100% methylated sites and nucleosome occupancy at CGI borders (A) Four representative examples showing the relative positions of the 100% methylation sites and within the CGI. (B) The enrichment level of stably maintained 100% methylated sites within 1500 bp around the CGI border. (C) Nucleosome occupancy within 1500 bp around the CGI border. In (B) and (C), positive values on the x-axis represent positions inside the CGI.

We also examined any correlations between these stably maintained fully methylated sites and histone modifications, since previous work has noted a link between DNA methylation and some forms of histone modification [ 7, 8] . In particular, we compared the high-fidelity 100% methylation sites with published H3K9me3, H3K27ac, H3K4me1, H3K4me3 and H3K79me2 ChIP-seq data (see Gene Expression Omnibus for GSM4955128, GSM3854054, GSM3374691, GSM945267 and GSM569089). However, we failed to identify any correlation with any of the known histone modifications ( Supplementary Figure S4). Hence, this lack of correlation suggests that maintenance of full methylation at these CpG sites is probably not a consequence (or cause) of specific histone modifications, and so must involve a mechanism that does not depend on these modifications.

With the relative rarity of fully methylated CpG sites that are stably maintained, an intriguing question that arises is whether at least some of these are cell-type specific. If so, their potentially functional consequences notwithstanding, these sites could serve as markers to identify specific cell types within a mixture of tissues with enormous practical applications. With the three different cell types, we examined CpG sites that were fully methylated in only one cell type and completely unmethylated (0%) in the other two cell types. In this way, we identified 24, 95, and 288 unique stably maintained fully methylated CpG sites in ARPE-19, SW1353, and Jurkat cells, respectively. Since our analysis here (RRBS) only included approximately 10% of all the CpG sites present in the human genome, the total number of such cell type-specific sites in the genome should indeed be much greater. Therefore, it is highly probable that unique identifiers could be found even when a much greater number of different cell types is considered. Perhaps such unique identifiers could also be discovered for certain cancer cells, thus allowing highly sensitive and early detection of these cells in liquid biopsies, a tantalizing possibility that requires further investigation. At a more basic level, such identifiers would also allow a quantitative analysis of constituting cell types with bulk analysis of tissues or even organs, providing a convenient alternative to demanding techniques such as single-cell analysis [ 9, 10] .

In summary, we introduced an RRBS-based quantitative method to identify, for the first time, faithfully maintained fully CpG methylated sites during cell passaging by combining high-depth sequencing and temporal profiling. We showed that these sites are only a fraction (as low as 2%) of the CpG sites that are normally considered methylated at the population level. This paucity strongly suggests unique mechanisms for their maintenance and functional significance, which can now be further investigated. We also showed that there were at least tens to hundreds of cell-type specific stably maintained fully methylated CpG sites that could be used as unique cell type identifiers. If this finding still holds true when a larger variety of cell types have been included (including those associated with disease states such as cancer), these sites might be more effective in the use of DNA methylation for diagnostic applications. We therefore postulate that further studies of these stably maintained fully methylated CpG sites will not only prove informative in understanding their role in cell functioning but also provide alternative and perhaps more effective clinical markers with much higher sensitivity and reliability than those presently available.

## Availability of Data

The datasets GSE176335 for RRBS data generated in this study can be found in the Gene Expression Omnibus (GEO): https://www.ncbi.nlm.nih.gov/geo/query/acc.cgi?acc=GSE176335.

The dataset GSE176336 for MNase-seq data generated in this study can be found in the Gene Expression Omnibus (GEO): https://www.ncbi.nlm.nih.gov/geo/query/acc.cgi?acc=GSE176336.

## Supporting information

309Supplementary_materials

## Supplementary Data

Supplementary data is available at *Acta Biochimica et Biophysica Sinica* online.
