# Supplementary material for: Temporal profiling with ultra-deep RRBS sequencing reveals the relative rarity of stably maintained methylated CpG sites in human cells: Rarity of stably maintained methylated sites in human cells
Source: Acta Biochim Biophys Sin (Shanghai). 2022 Dec 26;54(12):1935–8. doi: 10.3724/abbs.2022185 (PMC10157517; doi:10.3724/abbs.2022185)
Supplement: 309Supplementary_materials [file 309Supplementary_materials.pdf]

**Supplementary Materials**

**Materials and Methods**

**Cell culture**

ARPE-19 cells, SW1353 cells and Jurkat cells were purchased from the Cell Bank (Shanghai Institutes for Biological Sciences, Chinese Academy of Sciences, Shanghai, China). ARPE-19 and SW1353 cells were cultured with DMEM/F-12 medium (Biological Industries, Biet Haemek, Israel), and Jurkat was cultured with RPMI 1640 medium (Biological Industries), both containing 10% fetal bovine serum (Gibco BRL/Invitrogen, Carlsbad, USA), 100 μg/ml streptomycin, and 100 IU/ml penicillin, and incubated at 37°C and 5% $CO_2$. After 48 h during which the cells roughly doubled in number, half of the cells were collected and their DNA was extracted, while the remaining cells continued to culture. Following DNA replication in S phase, it is well known that there is a time delay before DNA methylation of the newly synthesized DNA, resulting in a significantly lower level of genomic DNA methylation in the S phase compared with other phases (G1/G0 and G2/M) [1]. Thus, to avoid methylation heterogeneity caused by some cells in S phase, we cultured cells to the 20$^{th}$ and 30$^{th}$ generations, and then conducted serum starvation for 24 h to synchronize the cells to the G0 phase before collecting the cells. The ARPE-19 and SW1353 cells were then desorbed from the culture dish by digestions with 0.25% Trypsin-EDTA (the Jurkat cells are suspended cells) (Thermo Scientific, Waltham, USA) and collected for DNA extraction.

**DNA extraction**

Each of the cells were washed twice with PBS and then collected by centrifugation. Genomic DNA was extracted from the cells by using the Axygen Genomic DNA Miniprep Kit (Axygen Scientific, Corning, USA) according to the manual. The DNA was quantified by Nanodrop One (Thermo Scientific) and the quality was evaluated by running 1.5% agarose gel electrophoresis.

### Reduced representation bisulfite sequencing

To evaluate the conversion error rate of bisulfite conversion, a methylated spike-in and an unmethylated spike-in were used. We chose two different regions from lambda DNA as the templates for the spike-ins. One template was amplified with the dNTP Mix to synthesize the unmethylated spike-in, and the other one was amplified with the 5-Methylcytosine dNTP Mix to synthesize the methylated spike-in. The spike-ins were then fragmented to about 300 bp by Covaris (Covaris, Woburn, USA). After constructing libraries using NEBNext Ultra II Library Prep Kit (New England BioLabs, Beverly, USA) for Illumina sequencing separately, the methylated spike-in and unmethylated spike-in were mixed together and then added to the genomic DNA at a ratio of 1% before bisulfite conversion.

Two micrograms of genomic DNA were used for *Msp*I (NEB, San Diego, USA) digestion, and the fragments were recovered by using the AxyPrep PCR Clean kit (Axygen, Suzhou, China). After constructing the sequencing library and mixing the spike-in, bisulfite conversion was performed on the libraries using the EZ DNA Methylation-Gold Kit (Zymo Research, Irvine, USA), which was also used to recovery the libraries. Epimark Hot Start Enzyme was used for PCR to amplify the libraries. To obtain the fragments exactly, vertical TBE-PAGE electrophoresis was performed on the 6% TBE-PAGE Gel (Invitrogen, Carlsbad, USA) to better separate the bands. After recovering the libraries from the gel, the Agilent 2100 Bioanalyzer system (Agilent, Santa Clara, USA) was used to confirm the quality of the library. High-throughput sequencing was performed on Illumina NovaSeq 6000 with paired-end 150 bp as the sequencing mode.

### Data analysis of RRBS

To determine the methylation level of the CpG sites in the RRBS data, we used Trim Galore with the rrbs parameter to apply adapter and quality trimming to the raw data, then used Bismark to align trimmed reads to the reference genome (assembly hg38),

followed by the application of a supplementary bismark_methylation_extractor script that operates on the Bismark output files and extracts the CpG methylation levels [2].

Unmethylated lambda DNA is used to estimate the conversion rate and methylated lambda DNA is used for estimating the inappropriate conversion rate. We mapped raw reads to the lambda DNA genome, removed PCR duplicates using bismark_methylation_extractor, then calculated the bisulfite conversion rate (that is, cytosine is not converted to uracil) and inappropriate conversion rate (that is, 5m-cytosine is converted to uracil) [3].

With this, the inappropriate conversion rate (ICR) is calculated as:

$$ICR = \frac{M_c}{M_{5mc} + M_c}$$

where the numbers of methylated cytosines and unmethylated cytosines detected in methylated lambda DNA are $M_{5mc}$ and $M_c$, respectively.

Similarly, the conversion rate (CE) is:

$$CE = \frac{U_c}{U_{5mc} + U_c}$$

where the numbers of methylated cytosines and unmethylated cytosines detected in the unmethylated lambda DNA are $U_{5mc}$ and $U_c$, respectively.

With these, the measured methylation level (*ml*) is given by:

$$ml = (CE - ICR) \times ml_p + 1 - CE$$

where $ml_p$ is the methylation level before bisulfite conversion.

To determine the statistical significance of the measured levels in methylation, we considered the detection of methylation as a sampling process, where the detected level of methylation is associated with the probability of a successful event and the sequencing depth is the number of independent experiments. As there are a large

number of cells that are sampled, the detection of the methylation level can be approximated by a binomial distribution.

In particular, the probability distribution of the methylation level is calculated as:

$$\mathrm{P}\left(ML=\frac{k}{n}\right)=\binom{n}{k}ml^{k}(1-ml)^{n-k} \qquad \text{, k=0, 1, 2, ...n}$$

where *ml* is the methylation level after bisulfite conversion, *n* is the sequencing depth of the CpG sites, and *k* is the number of methylated cytosines detected at this CpG site. Based on this relation, a *P*-value at a certain methylation level can be calculated, correcting using the Benjamini-Hochberg (BH) method [4].

Since this is a monoclonal culture, there are mainly three initial states for each CpG site in a single cell: 100% methylation, 50% methylation and 0% methylation. CpG sites with methylation levels other than 100%, 50% or 0% are defined as dynamic CpG sites. Taking the 100% methylation sites as an example, the null hypothesis is that the actual methylation level of these CpG site is 100%. In this work, a *P*-value≥0.1 was taken as the acceptable range for the null hypothesis, and it was considered that there was no detectable change in methylation at this site and, thus, the methylation level was considered as 100%.

Since smaller methylation changes can be detected with deeper sequencing depth, to obtain enough statistical power, CpG sites with coverage≥100-fold were only analyzed in this work, which we note is stricter than the cutoff of 10-fold in most prior work.

**MNase-seq and Nucleosome data**

About $5\times10^{5}$ ARPE-19 cells in interphase were lysed in 0.1% NP-40 buffer and then incubated with 20 U Micrococcal Nuclease (NEB, San Diego, USA) at 37°C for 5 min to digest the mono-nucleosomes [5]. The DNA was then incubated with RNase A for 1 h at 37°C, and further with 1% SDS and 20 μg/ml proteinase K (Thermo Scientific) for

2 h at 55ºC. After phenol extraction and ethanol precipitation, the DNA was separated by 1.5% agarose gel electrophoresis and the ~150 bp DNA fragments were recovered using a gel extraction kit (Axygen Scientific). The library was generated via the NEB Next Ultra II DNA library Prep kit for Illumina sequencing. The quality of library was confirmed by Agilent 2100 Bioanalyzer system.

**Genomic annotation**

The annotation of genomic features were based on the R package TxDb.Hsapiens.UCSC.hg38.knownGene. The distribution of CpG sites in genomic features (Supplementary Figure S2) was calculated with the R package ChIPseeker [6].

**Enrichment level of stably maintained 100% methylated sites and nucleosome occupancy score at the CGI border**

In order to verify the enrichment of stably maintained 100% methylated sites in the border of CGI, we conducted an overall analysis of CpG islands with an average methylation level greater than 10%, to avoid the interference of CGI with low methylation levels. We first selected the CpG islands with lengths greater than 1500 bp and used the CpG island border as the center point. Then, we obtained 750 bp upstream and downstream of the CpG island border, and divided it into 10 bins of 150 bp (the 5′ end and the 3′ end are processed in the same way and the upstream and downstream were respectively merged together). The level of enrichment in each bin was then calculated.

For the nucleosomal data, we take the midpoint of the sequenced fragment as the center position of the nucleosome, and obtain the position of the nucleosome occupancy at different positions in the genome. We mapped the raw MNase-seq data using bowtie2 to the reference genome (assembly hg38) and the midpoint of fragments with lengths

between 120 to 180 bp were smoothed. For the enrichment analysis, only CGIs with more than 10% methylation were examined.

## References


1. Charlton, J., et al., Global delay in nascent strand DNA methylation. Nat Struct Mol Biol 2018, 25: 327-332.
2. Krueger, F. and S.R. Andrews, Bismark: a flexible aligner and methylation caller for Bisulfite-Seq applications. Bioinformatics 2011, 27: 1571-1572.
3. Holmes, E.E., et al., Performance evaluation of kits for bisulfite-conversion of DNA from tissues, cell lines, FFPE tissues, aspirates, lavages, effusions, plasma, serum, and urine. PloS One 2014, 9: e93933.
4. Benjamini, Y. and Y. Hochberg, Controlling the false discovery rate: a practical and powerful approach to multiple testing. Journal of the Royal Statistical Society: series B (Methodological) 1995, 57: 289-300.
5. Pajoro, A., et al., Profiling nucleosome occupancy by MNase-seq: experimental protocol and computational analysis. Methods Mol Biol 2018, 1675: 167-181.
6. Yu, G., L.-G. Wang, and Q.-Y. He, ChIPseeker: an R/Bioconductor package for ChIP peak annotation, comparison and visualization. Bioinformatics 2015, 31: 2382-2383.

**Supplementary Table S1. Key parameters of RRBS data in Jurkat, ARPE-19 and SW1353 cells**

| Cell | Clone | Mapping efficiency | 120~220 bp | Conversion rate | Inappropriate conversion rate |
|---|---|---|---|---|---|
| Jurkat | clone1 G20 | 68.50% | 67.55% | 99.20% | 1.90% |
| | clone1 G30 | 68.60% | 65.88% | 99.20% | 1.80% |
| | clone2 G20 | 68.60% | 73.93% | 99.30% | 2.00% |
| | clone2 G30 | 72.90% | 71.73% | 99.20% | 2.30% |
| | clone3 G20 | 68.50% | 77.49% | 99.30% | 2.00% |
| | clone3 G30 | 76.90% | 69.06% | 99.20% | 2.10% |
| ARPE-19 | clone1 G20 | 59.30% | 75.69% | 99.10% | 2.10% |
| | clone1 G30 | 65.60% | 62.95% | 99.10% | 2.00% |
| | clone2 G20 | 65.40% | 73.52% | 99.00% | 2.10% |
| | clone2 G30 | 74.70% | 70.75% | 99.10% | 2.40% |
| | clone3 G20 | 66.90% | 66.88% | 99.00% | 2.20% |
| | clone3 G30 | 68.70% | 72.62% | 99.00% | 2.10% |
| SW1353 | clone1 G20 | 60.60% | 79.61% | 99.10% | 2.10% |
| | clone1 G30 | 63.50% | 81.27% | 99.10% | 2.40% |
| | clone2 G20 | 60.90% | 75.17% | 99.10% | 2.00% |
| | clone2 G30 | 67.60% | 73.20% | 99.00% | 2.10% |
| | clone3 G20 | 65.50% | 75.20% | 99.20% | 2.10% |
| | clone3 G30 | 68.00% | 63.69% | 99.00% | 2.00% |

**Supplementary Table S2. Basic characteristics of RRBS data in RPE, ARPE-19, T-cell and Jurkat cells** RPE and T-cell data are from the published data at GSM1063306 and GSM3732785. Methylated CpG sites were defined as CpG sites with a methylation level greater than 0.

| | RPE | ARPE-19 | | T-cell | Jurkat | |
|---|---|---|---|---|---|---|
| | / | G20 | G30 | / | G20 | G30 |
| CpG sites number (≥10×) | 715198 | 827078 | 651658 | 2321737 | 1832794 | 1949051 |
| Methylated CpG sites number (≥10×) | 581221 | 533138 | 456684 | 1694765 | 1521860 | 1542682 |
| Methylated CpG sites proportion (≥10×) | 81.27% | 64.46% | 70.08% | 73.00% | 83.03% | 79.15% |

**Supplementary Table S3. Classification of CpG sites in the ARPE-19, Jurkat, SW1353 cells.** All CpG sites were defined as CpG sites with depths of no less than 100-fold between the 20th and 30th generations.

| Generation | Items | ARPE-19 | | Jurkat | | SW1353 | |
|---|---|---|---|---|---|---|---|
| | | CpG site | Percentage | CpG site | Percentage | CpG site | Percentage |
| G20 | All CpG sites (≥100) | 1370430 | 100% | 1117190 | 100% | 1108253 | 100% |
| | ML=1 | 313457 | 22.87% | 144948 | 12.97% | 35511 | 3.20% |
| | ML=0.5 | 41922 | 3.06% | 46482 | 4.16% | 33009 | 2.98% |
| | ML=0 | 231851 | 16.92% | 192046 | 17.19% | 270669 | 24.42% |
| | Dynamic | 783200 | 57.15% | 733714 | 65.68% | 769064 | 69.39% |
| G30 | All CpG sites (≥100×) | 1370430 | 100% | 1117190 | 100% | 1108253 | 100% |
| | ML=1 | 150010 | 10.95% | 76749 | 6.87% | 34554 | 3.12% |
| | ML=0.5 | 41942 | 3.06% | 46565 | 4.17% | 33047 | 2.98% |
| | ML=0 | 232886 | 16.99% | 196297 | 17.57% | 322316 | 29.08% |
| | Dynamic | 945592 | 69% | 797579 | 71.39% | 718336 | 64.82% |

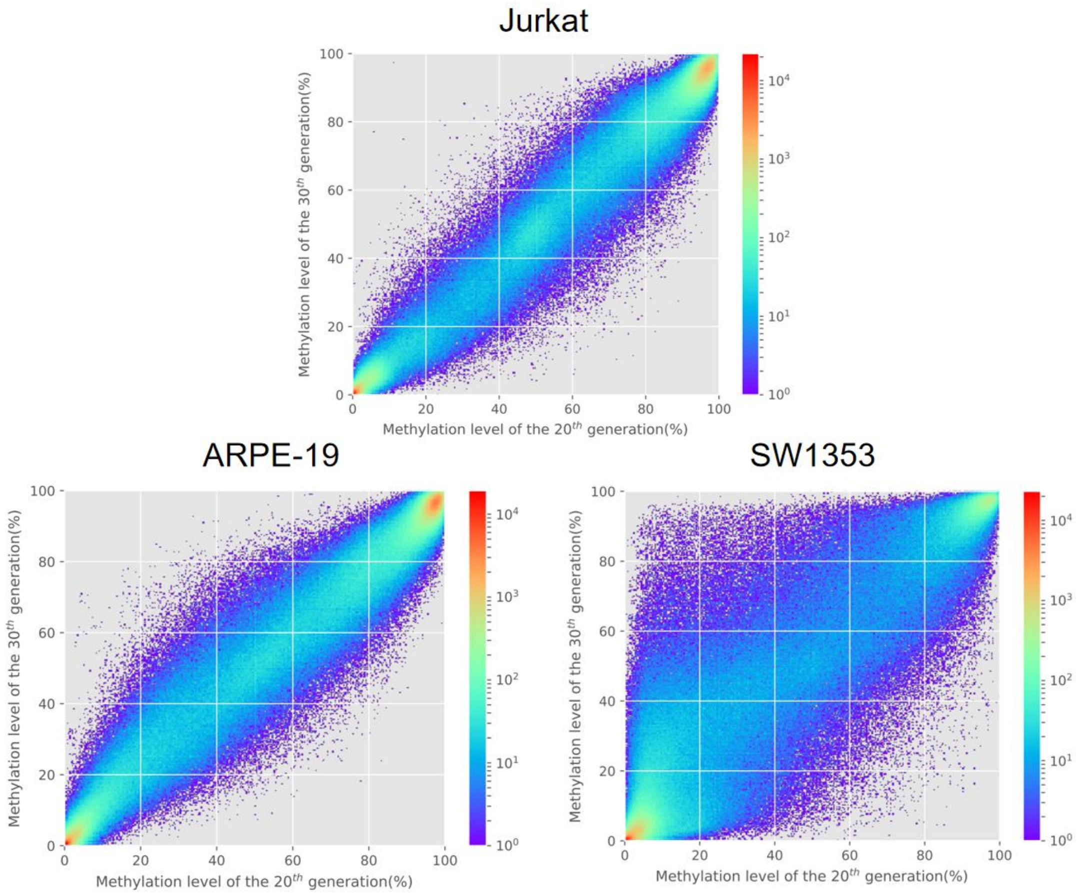


**Supplementary Figure S1. The distribution of methylation level of the $20^{th}$ generation and $30^{th}$ generation in Jurkat, ARPE-19 and SW1353 cells** Each point in the figure represents a CpG site with read coverage ≥100-fold.

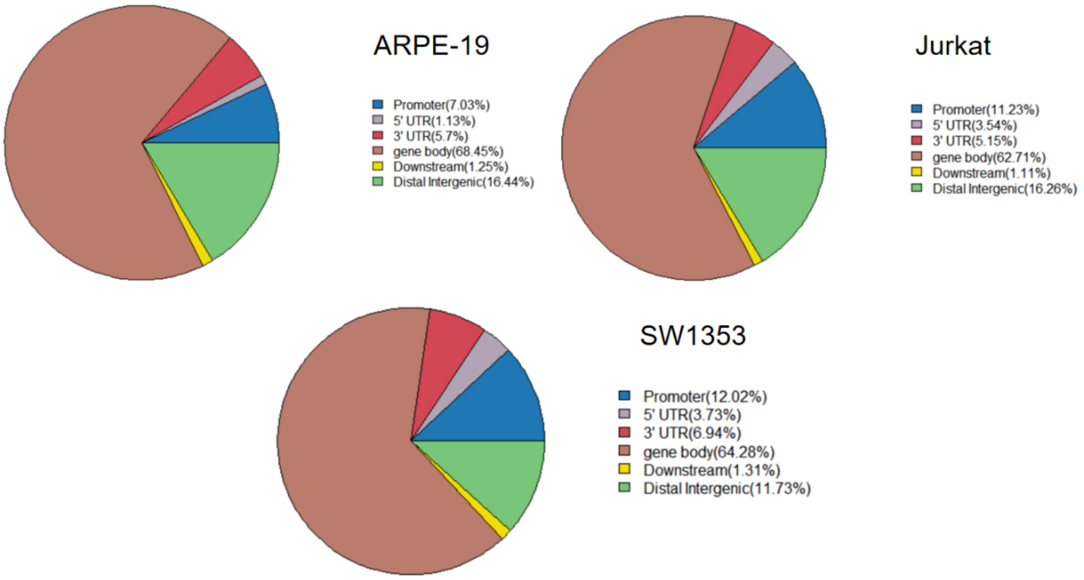


**Supplementary Figure S2. Distribution of the stably maintained 100% methylated sites in Jurkat, ARPE-19 and SW1353 cells in different annotated regions of the genome** The promoter was defined as 2 kb regions upstream of each transcript start site without overlap with any known gene body. The gene body was defined as the region that includes exons and introns. Downstream refers to 3 kb that downstream of each transcript end site.

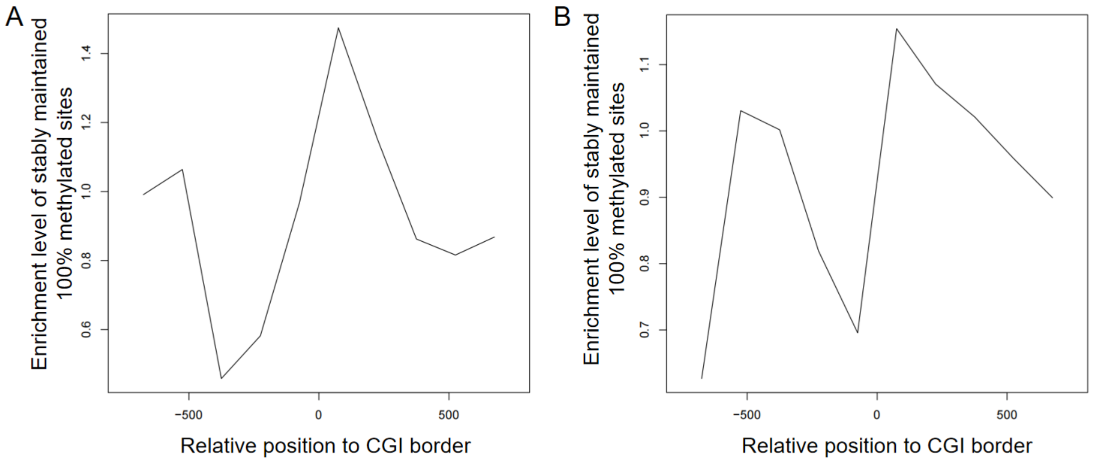


**Supplementary Figure S3. The enrichment of stably maintained 100% methylated sites at the CGI border in (A) SW1353 and (B) Jurkat cells**

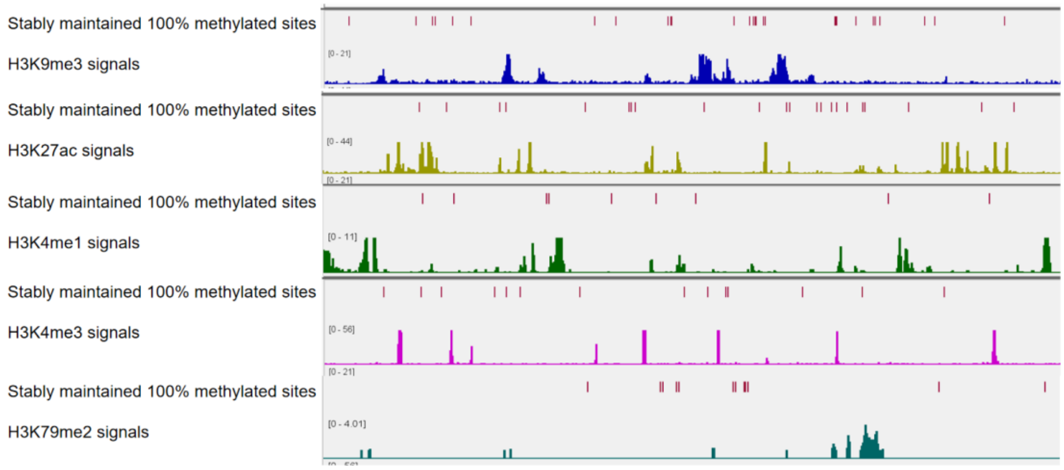


**Supplementary Figure S4. Examples of positional relationship between stably maintained 100% methylated sites and ChIP-seq signals**
